# How I treat rituximab refractory patients with WM

**DOI:** 10.18632/oncotarget.26411

**Published:** 2018-12-07

**Authors:** Maria Gavriatopoulou, Ioannis Ntanasis-Stathopoulos, Efstathios Kastritis, Meletios Athanasios Dimopoulos

**Affiliations:** Department of Therapeutics, Alexandra General Hospital, National and Kapodistrian University of Athens, School of Medicine, Athens, Greece

**Keywords:** Waldenstrom Macroglobulinemia, refractory, rituximab, ibrutinib, MYD88

Waldenstrom Macroglobulinemia is a B-cell low grade lymphoma characterized by bone marrow infiltration by monoclonal lymphoplasmacytic cells along with secretion of monoclonal IgM paraprotein [1]. Rituximab based regimens remain the gold standard for patients with symptomatic disease at all timepoints. However, the disease will eventually become resistant to rituximab, and therefore the need for novel treatment choices is more than imperative. MYD88L265 is a single activating somatic mutation identified in the majority of patients with WM [2]. MYD88 activates nuclear factor κB (NF-κB) through two different pathways which include Bruton's tyrosine kinase (BTK) and the interleukin-1 receptor-associated kinases (IRAK1 and IRAK4) [3]. Ibrutinib is a BTK inhibitor which binds to BTK irreversibly and selectively, therefore leading to apoptosis of WM cells that harbor the specific mutation. Ibrutinib was initially evaluated in a phase 1 study with very promising results and this provided the rationale for further investigation [3–4]. The efficacy and safety of ibrutinib was evaluated in a phase 2, open- label, multicenter trial. 63 patients with relapsed or refractory WM were enrolled and 62% of this patients population responded to treatment [5–6]. Based on these results on January 29, 2015, FDA approved ibrutinib (Imbruvica; Pharmacyclics) for the treatment of patients with symptomatic disease [7]. In order to confront the unmet need of rituximab refractoriness the iNNOVATE trial was designed in order to evaluate the efficacy and safety profile of ibrutinib monotherapy in patients refractory to rituximab. The results of the study demonstrated remarkable activity in this heavily pretreated population [8]. 31 patients with median age of 67 years were enrolled to receive 420 mg oral ibrutinib once daily until progressive disease or unacceptable toxicity. 13 (42%) out of 31 patients were classified as high risk as per the International Prognostic Scoring System for Waldenström Macroglobulinemia, all 31 patients were rituximab-refractory, while the median number of previous treatment regimens was 4. After 18 months of follow up the overall response rate was 90% and median time to first response 1 month. The estimated 18-month progression free survival and overall survival was 86% and 97% respectively. 2 patients discontinued treatment due to toxicity and the safety profile was well manageable. It is of high importance, that the proportion of rituximab­ refractory patients that responded to ibrutinib was very similar to what had been observed in non-refractory patients [5–6]. These encouraging results indicate that ibrutinib might overcome the poor prognosis related to rituximab refractoriness. The efficacy of ibrutinib does not seem to be affected by adverse prognostic features such as advanced age, increased levels of β-2 microglobulin, increased serum IgM levels, anemia and thrombocytopenia. It is indicated that *MYD88* and *CXCR4* mutational status might predict ibrutinib response, however due to the limited number of the patients these results should be evaluated extremely cautiously. The presence of CXCR4 mutation was correlated with slower responses, however the response rates remained high even in this subgroup. Progression-free survival was better for patients who had received one or two previous lines of treatment compared to the more heavily pretreated. The results from the randomized cohorts of the study will clarify the role of ibrutinib in earlier stages of the disease course and in the newly diagnosed setting. Despite the impressive results, we should not underestimate the financial burden of the drug combined with the indefinite administration until progression or unacceptable toxicity, especially if we compare it to chemoimmunotherapy which is completed in a definite time period with lower cost. This is the first study that provides data for rituximab refractory patients with WM, and certainly will become the rationale for further investigation. Other BTK inhibitors such as acalabrutinib and zanubrutinib are currently under investigation and might benefit this specific population in the future. Standard chemotherapy could be an alternative option; however, the low response rates and the induced toxicity limit its role especially for heavily pretreated patients with high tumor burden where rapid and deep responses are required. In conclusion, ibrutinib as a chemotherapy-free therapeutic approach will probably evolve to the novel standard of care for heavily pretreated rituximab refractory patients considering the sustained responses and prolonged progression-free survival, combined with the manageable toxicity profile.
